# Isolation of *Candida auris* from Ear of Otherwise Healthy Patient, Austria, 2018

**DOI:** 10.3201/eid2408.180495

**Published:** 2018-08

**Authors:** Shiva Pekard-Amenitsch, Agnes Schriebl, Wilhelm Posawetz, Birgit Willinger, Bettina Kölli, Walter Buzina

**Affiliations:** AGES–Austrian Agency for Health and Food Safety, Graz, Austria (S. Pekard-Amenitsch, A. Schriebl);; Private otorhinolaryngology practice, Graz (W. Posawetz);; Medical University of Vienna, Vienna, Austria (B. Willinger);; Medical University Graz, Graz (B. Kölli, W. Buzina)

**Keywords:** Candida auris, otitis externa, emerging yeast, Austria, yeast, fungi

## Abstract

The emerging pathogen *Candida auris* is isolated mostly from hospitalized patients and often shows multidrug resistance. We report on the isolation of this yeast in Austria from an outpatient’s auditory canal. The isolate showed good susceptibility against antifungals except for echinocandins; the patient was treated successfully with topical administration of nystatin.

The yeast *Candida auris* was first isolated from the external auditory canal of a person in Japan in 2009 (*1*). Reports from all continents except Australia (*2*) exist, but an outbreak in Europe was reported only recently, from a hospital in London (*3*). Major outbreaks of *C. auris* from countries in Europe have been reported from the United Kingdom and Spain, and sporadic isolations have been reported in Germany, France, Belgium, and Norway (*2*,*4*). We report on an isolation of *C. auris* in Austria, cultivated from an infection of the external auditory canal (otitis externa).

In January 2018, an otherwise healthy 22-year-old man came to an established otorhinolaryngology practice with therapy-refractory otitis externa in both ears that had persisted for almost 4 years despite antimicrobial drug treatment. The patient is an Austrian citizen with Turkish ancestry. He used to travel to Turkey frequently; his last visit was in 2017, and other trips abroad were not reported. A smear test was taken and sent to a microbiological laboratory for bacterial examination. A yeast grew after 48 hours at 37°C; the yeast was transferred to chromogenic mycological media, and an intense pink-colored yeast was cultivated on Brilliance Candida Agar (Oxoid, Basingstoke, UK). The isolate showed a pale rose color on BBL CHROMagar Candida Medium (Becton Dickinson, Heidelberg, Germany), and on Candida ID medium (bioMérieux, Marcy l’Étoile, France) (Technical Appendix Figure). On Sabouraud glucose agar and on malt extract agar (both from Becton Dickinson), the colonies were white. The maximum temperature at which the isolate grew on the chromogenic media and on malt extract agar was 42°C.

We further examined the isolate using the VITEK 2 system (bioMérieux). This system (version 08.01) identified the yeast as *C. auris* but with only 90% probability. We transferred the isolate to another laboratory and to the Austria national reference laboratory for antifungal susceptibility testing and molecular identification. Repeated examination with 2 more VITEK 2 systems with different versions (07.01, 99% probability *C. haemulonii*; 08.01, 98% probability *C. duobushaemulonii*) and the VITEK MS system (no identification) failed to identify the yeast correctly. Two different MALDI Biotyper Systems (Bruker Daltonics, Bremen, Germany) failed to identify the isolate with the in vitro diagnostic library. However, using the research use only library, we identified the isolate as *C. auris* with scores of 1.72 (formic acid extraction with additional washing step) to 1.96 (formic acid extraction) of a log(score) value between 0.00 and 3.00, which translates to a low confidence identification.

For molecular analysis, we extracted DNA from the isolate and performed PCR with the primer pairs ITS5 and ITS4 for the internal transcribed spacer region of the rRNA gene and NL-1 and NL-4 for the D1/D2 region of the large subunit of the rRNA gene (*5*,*6*). We then purified the amplicons and sequenced them with the same primers as for PCR. We submitted the sequence data of both sequences to GenBank under accession nos. MH071441 (ITS) and MH071440 (D1/D2). We deposited the strain for public use in the CBS-KNAW yeast collection of the Westerdijk Fungal Biodiversity Institute in Utrecht, the Netherlands, where it was assigned strain no. CBS 15366.

For antifungal susceptibility tests, we used 3 systems: the European Committee on Antimicrobial Susceptibility Testing (EUCAST) microdilution method (http://www.eucast.org/ast_of_fungi/), Micronaut (Merlin Diagnostica, Bornheim, Germany), and Etest (bioMérieux). Despite reports from other studies (*2*,*7*), our strain showed good antifungal susceptibilities for the tested antimycotics except for the echinocandins; their MICs were higher than those for the EUCAST breakpoints for *C. albicans*, for example. However, no breakpoints have been determined for *C. auris*. In the case of anidulafungin, Etest revealed a lower MIC than did the other tests. The susceptibility results (in MIC, μg/mL, by test) in detail are as follows: amphotericin B, 0.5–1.0 (EUCAST, Micronaut); anidulafungin, 0.012–0.125 (EUCAST, Micronaut, Etest); caspofungin, 0.032–0.125 (EUCAST, Micronaut); micafungin, 0.064–0.125 (EUCAST, Micronaut, Etest); 5-flucytosine, <0.064 (EUCAST, Micronaut); fluconazole, 0.25–0.5 (EUCAST, Micronaut, Etest); itraconazole, <0.03 (Micronaut, Etest); posaconazole, <0.008–0.032 (EUCAST, Micronaut, Etest); voriconazole, <0.008–0.016 (EUCAST, Micronaut, Etest); and isavuconazole, 0.002 (Etest).

We treated the patient twice weekly for 3 weeks with an oral suspension of nystatin; the patient did not return for the next 4 weeks. Thereafter, we examined the patient’s ears by otoscopy and found that the external ear canal was only slightly reddened and there were no signs of fungal growth.

In conclusion, *C. auris* is isolated not only from hospital settings and from severely ill patients but also from otherwise healthy persons. Despite new developments of databases and libraries for mass spectroscopy and biochemical-based instruments, molecular identification (e.g., ITS or D1/D2 domain of rDNA) is still the most, or in many cases the only, tool to identify *C. auris* reliably.

Technical Appendix Figure showing *Candida auris* colonies after 48 hours at 37°C on various chromogenic media.
