# Supplementary material for: Isolation of Candida auris from Ear of Otherwise Healthy Patient, Austria, 2018
Source: Emerg Infect Dis. 2018 Aug;24(8):1596–7. doi: 10.3201/eid2408.180495 (PMC6056136; doi:10.3201/eid2408.180495)
Supplement: Technical Appendix — Figure showing Candida auris colonies after 48 hours at 37°C on various chromogenic media. [file 18-0495-Techapp-s1.pdf]

Article DOI: https://doi.org/10.3201/eid2408.180495

Isolation of *Candida auris* from Ear of Otherwise Healthy Patient, Austria, 2018

Technical Appendix

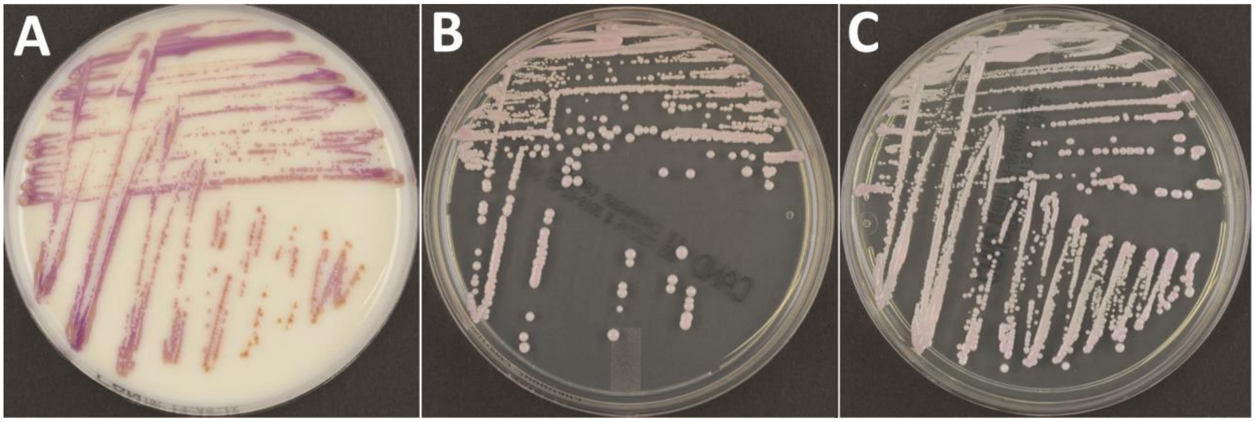

Technical Appendix Figure. *Candida auris* colonies from an otherwise healthy patient in Austria after 48 hours at 37°C on various chromogenic media: A) Brilliance Candida Agar; B) CHROMagar Candida; C) Candida ID.
